# Comprehensive Genetic and Morphological Analysis of Algerian Carob (*Ceratonia siliqua* L.) Accessions

**DOI:** 10.3390/plants14070990

**Published:** 2025-03-21

**Authors:** Oussama Smaili, Leonardo Paul Luca, Francesco Scollo, Nadjiba Chebouti-Meziou, Chiara Catalano, Mario Di Guardo, Gaetano Distefano, Elisabetta Nicolosi, Alessandra Gentile, Stefano La Malfa

**Affiliations:** 1 Laboratory of Soft Technologies, Valorization, Physicochemistry of Biological Materials and Biodiversity, Boumerdes University, Boumerdes 35000, Algeria; o.semaili@univ-boumerdes.dz (O.S.); n.chebouti@univ-boumerdes.dz (N.C.-M.); 2 National Seeds and Plants Control and Certification Center, Setif 19000, Algeria; 3 Department of Agriculture, Food and Environment (Di3A), University of Catania, 95123 Catania, Italy; leonardo.luca@unict.it (L.P.L.); fscollo@unict.it (F.S.); mario.diguardo@unict.it (M.D.G.); gaetano.distefano@unict.it (G.D.); elisabetta.nicolosi@unict.it (E.N.); alessandra.gentile@unict.it (A.G.); stefano.lamalfa@unict.it (S.L.M.)

**Keywords:** SSR markers, genotyping, phenotypic characterization, bioclimatic area, nuclear marker, chloroplast marker, structure analyses

## Abstract

Carob (*Ceratonia siliqua* L. Fabaceae) is a species of significant economic, ecological, and cultural importance in the Mediterranean region. It is valued for its adaptability to various environments and wide-ranging agricultural and industrial applications. Despite its potential, the genetic characterization of this species in Algerian territories has received little attention to date. The present study aims to decipher carob’s genetic structure and diversity in Algeria. This study presents a comprehensive morphological characterization of 39 Algerian carob accessions based on pod traits and molecular genotyping using eight nuclear and three chloroplast SSR markers across different geographical locations and environmental conditions. The morphological analysis revealed a discrete differentiation among accessions, primarily influenced by their area of origin. The genetic analysis identified 38 unique genotypes. Allelic richness indicated high polymorphism, with an average of 3.81 alleles and 5.36 genotypes for nuclear SSR markers. Chloroplast SSR markers showed lower variability but provided insights into population structure. Genetic analysis revealed distinct genetic clusters aligned with geographical and bioclimatic regions, supported by minimum spanning network analysis that showed the genetic flow patterns among accessions. Discriminant analysis of principal components identified five optimal sub-population groups, highlighting a genetic structure linked with different bioclimatic conditions. These findings evidence the complex genetic diversity of Algerian carob germplasm and offer valuable insights for the sustainable exploitation of carob genetic resources.

## 1. Introduction

The carob tree (*Ceratonia siliqua* L.) is a perennial, evergreen tree belonging to the Fabaceae family [1]. Although the exact origin of this species remains a subject of debate, a substantial amount of genetic and fossil evidence suggests a western Mediterranean origin [2,3,4]. It is a xerophilous species predominantly distributed in the Mediterranean semi-arid environments [5,6]. The carob tree is well adapted to regions characterized by mild, wet winters and extended, dry summers. It is widely cultivated in countries such as Spain, Italy, Turkey, and Greece, where it thrives in well-drained soils and demonstrates excellent tolerance to both heat and drought. Typically, the tree reaches a height of 5 to 10 m, occasionally ascending to 15 m under optimal conditions [7]. It features a broad, spreading canopy with a rounded, umbrella-like shape. The carob tree can be dioecious or hermaphroditic, producing small, numerous flowers that are spirally arranged along the inflorescence axis in raceme clusters resembling catkins. These flowers grow on short spurs from old wood and even directly on the trunk (cauliflory). Pollination occurs through both wind and insects [8]. Carob possesses a deep and expansive root system that allows it to anchor in dry, rocky soils and access deep groundwater sources during prolonged dry periods. This extensive root structure is vital for its survival in arid climates. Its robust structure and resilience in harsh climates contribute to its popularity in agroforestry and landscaping. The fruits, are large, flattened, leathery pods that can grow up to 30 cm in length and contain sweet, edible pulp. [3,9].

In the Mediterranean regions, this tree is considered a true natural treasure [5,6]. This species has demonstrated notable resilience, vigor, and ability to thrive in extreme environmental conditions; also, the species supports various industrial applications, ranging from the food to the pharmaceutical sector [10]. Carob cultivation is of considerable interest to the food industry, primarily due to its applications in the production of syrups and molasses. Furthermore, carob flour is widely regarded as an excellent substitute for cocoa in creating an array of baked delicacies [11,12]. In addition, carob is acknowledged as a medicinal plant due to its extensive array of health benefits, which are well-documented in various studies [13,14,15].

Numerous studies have been conducted to analyze the genetic diversity of carob trees in the Mediterranean regions. Researchers have utilized various molecular markers in their investigations, including AFLP [16], RAPD and AFLP [17], EST-SSR [18], and SSR markers [19]. Additionally, the molecular variability in both wild and natural populations of carob trees has been explored using RAPD markers [20,21]. A more recent study conducted in 2022 [4] advanced our understanding of the carob tree’s evolutionary history and domestication. The researchers used over 1000 microsatellite genotypes to delimit seven carob evolutionary units and 3557 single nucleotide polymorphisms (SNPs) generated by restriction-site associated DNA sequencing (RADseq) to investigate genome-wide diversity, shedding light on the carob tree’s genetic structure and evolutionary patterns [4].

Given its economic value as a source of edible pods and its potential as a substitute for food and pharmaceutical products, extensive efforts have been devoted to characterizing and understanding the morphological diversity of carob cultivars, especially in relation to fruit [6]. The morphological characteristics of the carob fruit, particularly its size, shape, color, and pulp quality, are of prime interest in these studies [11,15]. Since carob fruit is the primary commercial product of the tree, with its pulp used in food production, variations in this biochemical composition can directly affect both the economic viability and marketability of different cultivars. For instance, fruit size and thickness are critical factors that determine yield and the quality of the pulp as a raw material for processing. Larger pods with a higher pulp-to-seed ratio are often preferred, resulting in higher economic returns due to the increased quantity of edible products [5,22,23,24]. However, more recently, seeds (from which Carob Bean Gum, CBG, is obtained) represent a valuable product since CBG is an appreciated thickener agent used in the food industry [11].

Despite the growing interest in the genetic diversity of carob and the increasing attention to this field by researchers, farmers, and the Algerian Ministry of Agriculture in recent years, no specific genetic study has been conducted in Algeria. Several studies have been conducted in Algeria to examine the local carob germplasm’s phenotypic diversity and nutritional composition. These studies have contributed to a better understanding of the various characteristics of carob populations present in the country [6,22,23,24,25,26,27,28,29,30]. In this context, the present study aims to investigate Algeria’s carob tree’s genetic and morphological diversity. This approach represents a prerequisite for developing conservation and management strategies, particularly in the context of challenges posed by climate change and the significant genetic erosion affecting the Mediterranean region [18,19]. In order to achieve this objective, a targeted survey was conducted in various areas of Algeria. The aim of the investigation was to identify and characterize carob accessions growing in diverse natural or agricultural habitats and to gather information for the in situ conservation of this iconic species by identifying the most diversified and best-adapted accessions to different ecological conditions. The selected accessions were characterized using various morphological traits, and the genetic identity and level of variation among these carob accessions were then assessed using a panel of selected SSR markers [16,18,19]. Moreover, the carob accessions were analyzed by considering their bioclimatic region of origin as the main genetic strata, as this approach allows a deeper understanding of how environmental factors shape the genetic diversity within the species. By linking the genetic structure of the accessions to their respective bioclimatic regions, this study aims to identify patterns of adaptation to specific environmental conditions, providing valuable insights into the evolutionary processes and potential for future breeding programs. Overall, the main objectives of the study were to genotype carob trees in Algeria, determine the relationships and structure among these accessions, and assess the correlation between genetic and morphological data.

## 2. Materials and Methods

### 2.1. Plant Material

The carob germplasm collection used in this study comprised 39 accessions encompassing different Algerian bioclimatic areas, incorporating a diverse range of environments from wetlands to arid regions. It includes humid, sub-humid, arid, and semi-arid zones, with altitudes varying from 33 to 995 m above sea level. Regarding productive status, 38 female accessions and 1 hermaphroditic accession were sampled. This plant material comes from 17 different regions of Algeria. Information about the origin, sex, and status of each accession is specified in Table 1.

### 2.2. Morphological Characterization

To evaluate the morphological diversity within the studied carob collection, twelve pod and seed parameters were measured, following methodologies outlined by Mahdad et al. (2022) and Smaili et al. (2024) [6,24]. The pods were harvested from July to September 2024. For each accession, thirty pods were randomly selected. Following pod dehiscence, a single seed was extracted from each pod for a final sample size of thirty seeds per accession. The traits that have been recorded are pod length, pod width, pod thickness, pod weight, number of seeds per pod, seed weight, pulp weight, seed length, seed width, seed thickness, seed yield, and pulp percentage.

### 2.3. Molecular Analysis

#### 2.3.1. DNA Isolation

Leaves were collected in June 2024 from different sides of the canopy of each accession and bulked together. Therefore, one sample was collected for each analyzed accession. The DNA extraction was performed on 100 mg of young leaflets, which were stored in silica gel to maintain tissue integrity and prevent degradation, as described by Wilkie et al. [31]. The genomic DNA was isolated using a modified CTAB (Cetyltrimethylammonium Bromide) protocol based on Doyle and Doyle’s (1987) methodology [32]. Mechanical lysis of the leaf tissue was carried out using a TissueLyser II (Qiagen, Hilden, Germany) at 30 Hz for 30 s. A volume of 550 µL of a lysis buffer solution containing 3% CTAB, 0.2% β-mercaptoethanol, and 1% Polyvinylpyrrolidone (PVP) (Sigma Aldrich, St. Louis, MO, USA) was added. The samples were incubated at 65 °C for 15 min. Following incubation, samples were vortexed and centrifuged at 14,000 rpm for 8 min. The supernatant was carefully recovered and combined with 300 µL of chloroform isoamyl alcohol (24:1 *v*/*v*) (Sigma Aldrich) for purification. An additional centrifugation step was performed at 14,000 rpm for 8 min. The aqueous supernatant was then recovered and treated with 50 µL of ammonium acetate and 500 µL of pure ethanol (100%) to precipitate the DNA (Sigma Aldrich). The sample was incubated on ice for 20 min, then centrifuged to recover the precipitated DNA. After removing the supernatant, the DNA pellet was washed with 600 µL of 70% ethanol (Sigma Aldrich). Finally, the DNA was dissolved in 50 µL of ultra-pure water (Sigma Aldrich). The quality and quantity of the extracted DNA were verified by NanoDrop 2000 spectrophotometric measurements (Thermo Scientific, Waltham, MA, USA).

#### 2.3.2. SSR Analysis

Genotyping was performed using eight nuclear SSR (Simple Sequence Repeat) markers and the analysis of three chloroplast SSR markers of locus matK4LF in accordance with the work of Di Guardo et al. (2019) [19]. A working DNA solution of 30 ng/µL for each sample was prepared and stored at −20 °C until PCR analysis. The amplification of the eight SSRs was conducted in 15 μL volumes, comprising 30 ng of genomic DNA, 1X NH4 reaction buffer (Meridian Bioscience, Cincinnati, OH, USA), 0.2 mM dNTPs (Meridian Bioscience), 1 mM MgCl_2_, 0.167 nM of forward and reverse primers, labelled with a 0.13 nM of M13F fluorescent dye (FAM, PET, NED, or VIC; Eurofins Scientific, Luxembourg), and 1 U of Taq Polymerase (Meridian Bioscience). Amplification was performed using a PCR system Mastercycler Nexus GX2 (Eppendorf, Hamburg, Germany). The initial denaturation was carried out at 94 °C for 5 min, followed by 40 cycles of 94 °C for 30 s, 56 °C for 30 s, and 72 °C for 60 s, with a final extension at 72 °C for 30 min. The PCR products were analyzed through capillary electrophoresis, utilizing the services provided by Eurofins Genomics company (Eurofins Scientific, Luxembourg). The electropherograms were then analyzed using the Microsatellite Analysis app (Thermo Fisher Scientific).

### 2.4. Statistical Analyses

The morphological data were analyzed within the R software environment (v 4.4.2) [33] utilizing the “dplyr” package [34] to compute mean and standard deviation values. The principal component analysis was conducted with the “factorMineR” package [35]. An analysis of variance (ANOVA) the effect of bioclimatic strata factor was conducted using R software. Prior to genetic analysis, the germplasm was screened for duplicated multilocus genotypes across specified population strata using the “clonecorrect” function of the “poppr” R package [36]. The Cervus software (3.0.7) was utilized to assess the SSR genetic information, including calculating major allele frequency, the count of identified genotypes, the number of alleles at each locus, and both observed and expected heterozygosity (H_o_ and H_e_). The polymorphism information content (PIC) was also assessed using Cervus software [37]. Genetic data were further employed to generate a genotypic accumulation curve and calculate the Simpson index, utilizing the “poppr” R package [36]. Neighbor-joining analyses were performed using the “ape” package [38] based on the Prevosti distance matrix, calculated from the genetic data using the “poppr” package. A minimum spanning network was generated by a distance matrix using the “poppr.msn function”, and the resulting image was produced with the “plot_poppr_msn” function of the “poppr” R package. A discriminant analysis of principal components (DAPC) was performed to evaluate genetic stratification within the carob collection. The “adegenet” package in the R environment was used to determine the optimal number of K-means clusters based on the Bayesian Information Criterion. The dapc function from the “adegenet” package was applied to the genetic data, and posterior membership probabilities were visualized to illustrate genetic stratification according to the bioclimatic area of origin for the various carob accessions [39]. The Analyses of the Molecular Variance was conducted using the “poppr” R package. Figures for the strata analyses were generated using the “ggplot2” R package [40]. Finally, in order to investigate a possible correlation between phenotypic and genotypic data a Mantel test was conducted using the R package “vegan” [41].

## 3. Results

### 3.1. Morphological Analysis

**Figure 1:**
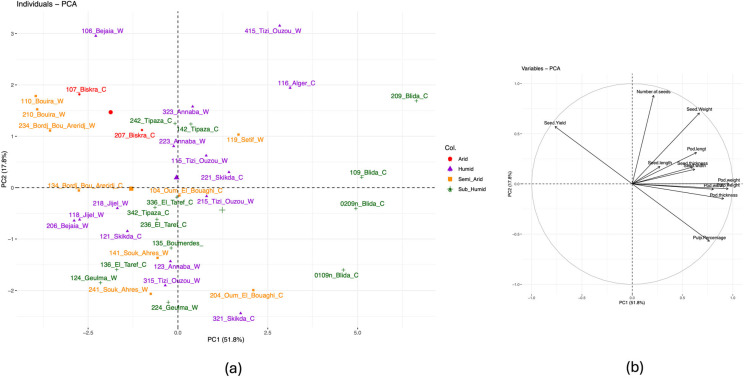
Principal component analysis of morphological traits of carob pods within the studied germplasm. (**a**) Distribution of individual pods, with color distinctions representing their respective bioclimatic area of origin. (**b**) Contribution of individual variables to overall variance observed in the analysis.

The principal component analysis (PCA) indicated a discrete pod morphological differentiation among the Algerian carob accessions evaluated in this study. The first two principal components collectively accounted for 69.6% of the total variance, with PC 1 contributing 51.8% and PC 2 contributing 17.8% (Figure 1). The distribution of individuals along these components showed distinct clustering patterns, influenced mainly by their region of origin. Accessions from the Blida region exhibited a distinct clustering pattern, which was similarly observed among accessions from other regions, albeit to a lesser degree (Figure 1a). The variable correlation plot (Figure 1b) highlighted the key traits contributing to the observed variation. The variables that contributed the most to the variability were the seed yield and pulp percentage (Pearson correlation coefficient = −1), the number of seeds, and the seed weight (Pearson correlation coefficient = 0.75). Supplementary Table S1 reports all the mean measures and standard deviations of the phenotypic trait analyzed.

Specifically, the pod traits exhibit considerable variation across the samples. The average pod length ranged from 112 to 201 mm, width varied between 15.9 and 26.52 mm, and thickness from 4.16 to 12.06 mm. Pod weight also showed significant variation, from 5.03 g to 37.23 g. The pulp weight and the percentage of seed and pulp content in each pod differed among samples, reflecting evidence of discrete variability.

For seed traits, the seed length ranged from 7.45 to 10.75 mm, seed width ranged from 5.84 to 7.68 mm, and thickness ranged from 3.21 to 4.62 mm. Seed weight per pod varied between 1.03 g and 3.22 g, indicating notable differences in seed size and weight.

The highest-performing sample, 615, stands out due to its remarkable pod and seed traits. It revealed a pod length of 181 mm, a width of 29.7 mm, and a thickness of 12.35 mm, with a total pod weight of 44.97 g. The seed weight per pod was 3.79 g, with individual seeds measuring 6.90 mm in length, 5.89 mm in width, and 4.06 mm in thickness. The pulp weight per pod is 41.18 g, accounting for 91.61% of the total pod weight. This sample excelled in both pod size and the proportion of pulp.

Conversely, the lowest sample, 0106, featured smaller pod and seed dimensions. Its pod length measured 141 mm, with a width of 20.7 mm and a thickness of 8.36 mm, resulting in a total pod weight of 22.31 g. The seed weight per pod was 2.16 g, with seeds measuring 6.23 mm in length, 5.38 mm in width, and 3.45 mm in thickness. The pulp weight per pod was 20.15 g, constituting 90.18% of the total pod weight.

The morphological analysis of carob pods and seeds across four bioclimatic regions (arid, humid, semi-arid, and sub-humid) reveals significant variations in certain traits. In contrast, others remain statistically non-significant (Supplementary Table S2). Below is a summary of the main findings. ANOVA results reveal significant differences in pod length and width across bioclimatic regions (*p* < 0.05 and *p* < 0.01, respectively). The sub-humid region produces the longest (164 mm) and widest (23.1 mm) pods, significantly larger than those in the humid (141 mm, 21.3 mm), semi-arid (140 mm, 19.4 mm), and arid (151 mm, 15.8 mm) regions. In contrast, pod thickness, pod weight, seed traits (count, weight, length, width, thickness), and seed yield show no significant differences (*p* > 0.05), indicating their stability across climates. These results suggest that more humid environments significantly influence pod size, while seed traits remain stable regardless of climate.

### 3.2. Genetic Analysis of the Carob Collection

The 39 Algerian carob accessions were genotyped with eight nuclear SSRs and three chloroplast genome loci. The genotyping analysis allowed the detection of one putative clone (Sample ID 109n) that was subsequently excluded from the analysis, leading to a final number of 38 unique accessions. A total of 37 alleles were identified within nuclear genome SSRs, while eight alleles were observed in the chloroplast genome SSRs. The allelic richness estimation allowed efficient discrimination of 97.7% of the samples, as shown by the accumulation curve (Supplementary Figure S1). No SSR markers displayed a Minimum Allele Frequency (MAF) less than 0.05. The mean number of alleles per loci and genotypes were 3.81 and 5.36, respectively (Table 2 and Table 3). As regards the nuclear SSR markers (Table 2), the AT15 marker showed the highest allelic and genotypic variability (eight alleles and 11 unique genotypes). The markers CTTT7 and TA7 displayed the lowest allelic and genotypic variability (two alleles for both and two and four genotypes, respectively). The mean H*_e_* and H*_o_* across the eight nuclear SSRs were 0.45 and 0.42; the highest H_o_ value was recorded for the TTA7 marker (0.82) and the lowest for the CTTT7 marker (0.21), and as regards the H_e_ values, the highest was the AT15 SSR marker with 0.63 and the lowest the CTTT7 marker with 0.19. Consequentially, the CTTT7 marker recorded the lowest PIC and Simpson index (0.17 and 0.2 D), the AT15 marker recorded the highest PIC value (0.59), and the AT15 and AT9 markers recorded the highest Simpson index value (0.62 D).

Regarding the chloroplastic genome SSR markers (Table 3), the observed variability was notably lower, as expected, likely due to their haploid nature and inherent conservativeness. Despite this limited variability, the data offers significant insights into the carob accessions diversity, as reflected in the relatively high Simpson index (0.85–0.86 D) compared to the nuclear SSR marker. The SSR profiles of all studied accessions are summarized in Supplementary Table S3.

### 3.3. Clustering Patterns and Genetic Relationships Among Studied Carob Accessions

**Figure 2:**
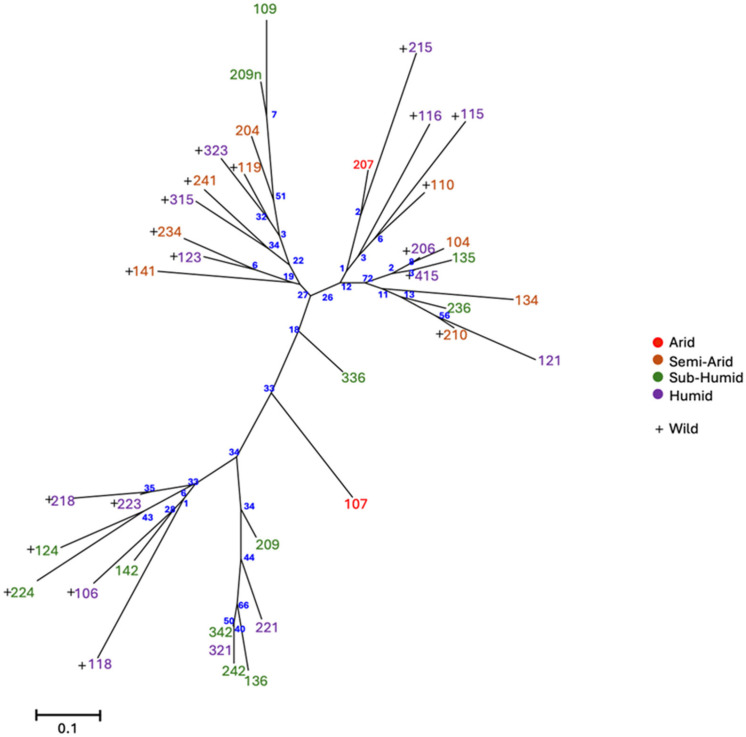
Unrooted neighbor-joining dendrogram of the 38 carob tree accessions, with bootstrap support values (blue numbers). Accessions are color-coded based on their bioclimatic area of origin: red for arid region, yellow for semi-arid region, green for sub-humid region, and violet for humid region. Wild accessions are marked with a cross symbol.

The neighbor-joining analyses conducted on the Prevosti distance matrix of the studied accessions revealed a broad range of pairwise distances, indicating significant genetic variability within the dataset. The lowest genetic distance values were observed between closely related accessions, such as 124 and 224, both wild genotypes of the same region (Guelma), which cluster together in the dendrogram (Figure 2) with minimal branch lengths, suggesting high genetic similarity.

The dendrogram reveals the presence of distinct clades, with accessions clustering according to their genetic similarity. One well-defined clade includes 124 Guelma, 224 Guelma, 223 Annaba, and 218 Jijel, characterized by relatively short branch lengths, suggesting a shared genetic background, potentially due to geographical proximity or limited genetic divergence. Another prominent clade encompasses accessions such as 236 El Taref, 134 Bordj Bou Arréridj, and 210 Bouïra, which exhibit moderate genetic distances from each other A third distinct clade contains accessions such as 104 Oum El Bouaghi, 135 Boumerdès, and 206 Béjaïa, with longer branch lengths, suggesting a higher level of genetic differentiation.

Additionally, the results show that most accessions (twelve accessions) from humid and sub-humid bioclimatic areas have diversified by branching together.

**Figure 3:**
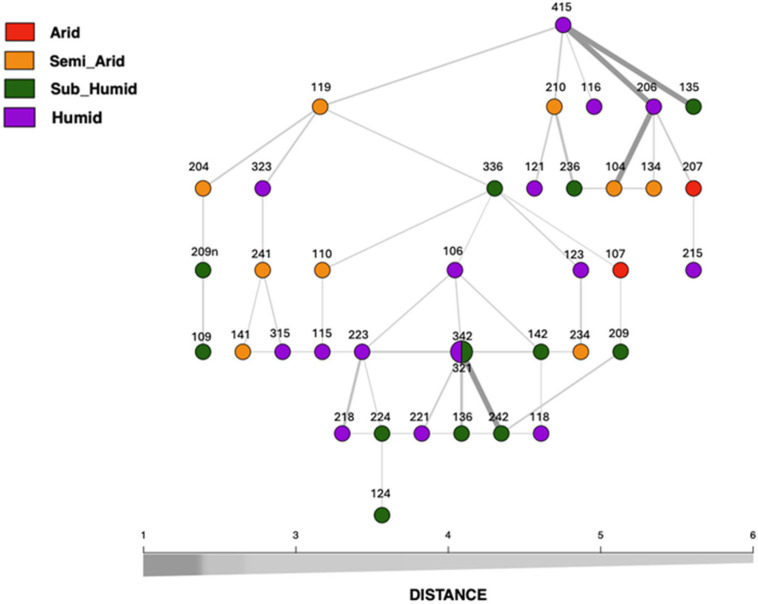
Minimum spanning tree constructed based on the dissimilarity distance of carob accessions, which are grouped and color-coded according to their bioclimatic area of origin. Color scheme is as follows: red for arid region, yellow for semi-arid region, green for sub-humid region, and violet for humid region. In this figure, thicker and darker edges represent shorter distances.

The minimum spanning network (MSN) analysis depicted (Figure 3) the genetic relationships among the 38 accessions. The network structure exhibited clustering patterns aligned with the four bioclimatic ecological classifications: semi-arid, humid, arid, and sub-humid. The color-coded visualization indicated that most accessions clustered within their respective bioclimatic groups, though some interconnections suggested genetic exchange or shared ancestry across different environments.

Several accessions played a central role in the network structure. Accession 119 Sétif was a key node, connecting to accessions 204 Oum El Bouaghi, 323 Annaba, 336 El Taref, and 415 Tizi Ouzou, reinforcing its strong genetic ties within the humid population. Similarly, accession 223 Annaba formed a central hub, linking 142 Tipaza, 218 Jijel, 224 Guelma, 321 Skikda, and 342 Tipaza, emphasizing its genetic connectivity across carob accessions. Notably, 321 Skikda and 342 Tipaza, represented by the same node due to their strong genetic likeness, were positioned at the intersection of the arid and semi-arid groups, indicating a potential genetic bridge between these environmental categories.

Some accessions exhibited substantial genetic similarity, as highlighted by thick edges in the network. For instance, the connection between 241 Souk Ahras and 315 Tizi Ouzou was robust, indicating a high degree of genetic similarity. Likewise, accessions 106 Bejaia and 142 Tipaza were closely linked, suggesting shared ancestry or recent genetic exchange. On the other hand, thinner edges represented more distant relationships, reflecting genetic divergence.

The positioning of accessions 116 Algiers and 118 Jijel in the network further confirmed their genetic differentiation. Accession 116 Algiers was directly linked to 415 Tizi Ouzou, while 118 Jijel was connected to 218 Jijel, reinforcing their distinct genetic trajectories. Furthermore, semi-arid carob accessions exhibited genetic connectivity with other ecological groups, mainly through accessions 321 Skikda and 223 Annaba, which bridged different population clusters.

### 3.4. Genetic Structure Analyses

**Figure 4:**
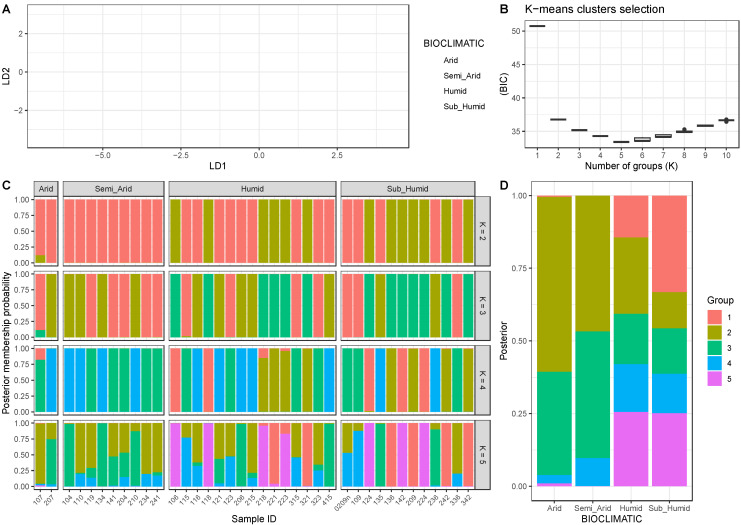
A discriminant analysis of principal components (DAPC) scatter plot on the genetic dataset is displayed in (**A**). Each point represents an individual projected onto the first two discriminant functions, LD1 and LD2. Colors indicate bioclimatic area classification: arid is shown in red, semi-arid in orange, humid in violet, and sub-humid in green. In (**B**), the plot shows Bayesian Information Criterion (BIC) values for different numbers of clusters (K) tested during K-means clustering analysis. (**C**,**D**) show results of structure analyses. The panel shows the posterior membership probabilities of individuals assigned to different clusters for K = 2, 3, 4, and 5. Each bar represents an individual, grouped by bioclimatic classification (arid, semi-arid, humid, sub-humid). Colors indicate different genetic sub-population group clusters. Panel D shows a bar plot of posterior membership probability from each bioclimatic category (arid, semi-arid, humid, sub-humid) assigned to different genetic clusters. Colors represent genetic sub-population groups.

The discriminant analysis of principal components (DAPC) distinguished among selected strata populations from humid, semi-arid, and arid bioclimatic areas (Figure 4A). Accessions from humid and sub-humid areas formed a cohesive cluster, indicating a shared genetic background. In contrast, the positioning of accessions from arid and semi-arid regions suggests a more structured genetic background with minimal overlap. Accessions from humid environments showed greater dispersion across LD1 and LD2.

The Bayesian Information Criterion (BIC) analysis results (Figure 4B) consistently identified K = 5 as the optimal number of genetic clusters, revealing a strong correspondence between genetic variation, geographic distribution, and bioclimatic conditions.

The BIC analysis provided an objective measure for determining the best-supported number of clusters. The BIC values decreased progressively from K = 1 to K = 5, with the lowest BIC value recorded at K = 5 (33.41). Beyond this point, the decrease in BIC was marginal, indicating that additional clusters would not significantly improve the model fit. This pattern suggests that five distinct groups best explain the genetic diversity within the dataset.

The genetic structure analyses (Figure 4C,D) confirmed a cohesive genetic configuration based on the bioclimatic area of origin. At K = 2, the accessions are broadly divided into two genetic cluster groups, reflecting a coarse division with limited differentiation. The accessions adapted to the Arid area show the predominance of sub-population Group 1 (in red) with a posterior membership probability percentage of 94.2% and a minor presence of sub-population Group 2 (in yellow) with 5.8%. The semi-arid bioclimatic population group showed 100% of the cluster Group 1. In relation to the two other bioclimatic areas of origin for the accession studied, the structure analysis for K = 2 reveals that in the humid area, Group 1 has a posterior membership probability percentage of 60%, while Group 2 has 40%. In the sub-humid area, the posterior membership probability percentage is 42% for Group 1 and 58% for Group 2.

As K increases, sub-structuring becomes more evident. For K = 3, the groups within the arid area show the following distribution: Group 1 accounts for 44.2%, Group 2 for 50%, and Group 3 for 5.8%. In the semi-arid area, the population is structured in two groups, with Group 1 comprising 55% and Group 2 45%. In the humid and sub-humid areas, the percentages are as follows: in the humid area, Group 1 represents 26.5%, Group 2 is 33.5%, and Group 3 is 40%; while in the sub-humid area, Group 1 is 25%, Group 2 is 16.7%, and Group 3 is 58.3%.

In the arid area, three principal sub-populations are identified for K = 4: Group 1 represents 8.8%, Group 3 constitutes 41.1%, and Group 4 is 50.1%; in the semi-arid area, two sub-populations are recognized: Group 2 accounts for 55.6% and Group 4 comprises 44.5%. In the humid and sub-humid areas, the distributions of the sub-population groups for K = 4 are as follow: in the humid area, Group 1 is 14.6%, Group 2 is 25.4%; Group 3 is 26.7%, and Group 4 is 33.3%; in the sub-humid area, Group 1 is 25%, Group 2 is 33%, Group 3 is 25%, and Group 4 is 17%.

At K = 5 (Figure 4D), the highest resolution for dissecting genetic structure reveals five distinct genetic sub-population groups. These groups align with the variation of bioclimatic zones, though some accessions exhibit different degrees of admixture. For the arid accessions, the distribution across sub-population groups is as follows: Group 1 represents 0.5%, Group 2 encompasses 60.2%, Group 3 encompasses 35.5%, Group 4 accounts for 2.9%, and Group 5 is 0.9%. Three sub-population groups are delineated in the semi-arid category: Group 2 comprises 46.9%, Group 3 accounts for 43.7Tab%, and Group 4 is 1%. Within the humid bioclimatic area, five sub-population groups emerge: Group 1 is 14.4%, Group 2 is 26.3%, Group 3 is 17.3%, Group 4 is 16.4%, and Group 5 is 25.6%. Lastly, the sub-humid bioclimatic area also accommodates five distinct sub-population groups: Group 1 totals 33.3%, Group 2 is 12.4%, Group 3 stands at 15.6%, Group 4 is 13.5%, and Group 5 reaches 25.1%.

The results of the AMOVA (Supplementary Figure S2) indicate a significant genetic differentiation between bioclimatic populations (Φ_CT = 0.041, *p* = 0.041), confirming the results of the DAPC structure analyses performed. This analysis also highlighted the genetic variation found within individuals (*p* = 0.001). This suggests that while there are notable differences between bioclimatic areas of origin, the majority of genetic diversity exists at the individual level. Furthermore, there is significant variation among individuals within the bioclimatic area of origin (*p* = 0.001), emphasizing that the genetic differentiation within populations is also considerable. These findings illustrate that both individual variation and population differentiation play important roles in shaping the genetic structure of the studied groups.

To evaluate the potential correlation between the phenotypic and genotypic data, a Mantel test was conducted. The analysis results indicated no correlation, with a Mantel statistic of r = 0.04 and a *p*-value of 0.2.

## 4. Discussion

The carob tree, long perceived as undervalued and lacking in commercial significance, is a crop with the potential to become an important agricultural and industrial resource for many countries in the Mediterranean region. Despite this potential, research has primarily overlooked its characterization, and current varietal catalogues do not fully capture the biodiversity of this tree crop in the Algerian territories [6]. The findings presented in this study make a substantial contribution to the understanding of the genetic structure, diversity, and relationships among carob tree genotypes across diverse Algerian geographical locations and environmental conditions. Through the analysis of genetic markers, this research contributes to a deeper knowledge of the genetic resources of this neglected species, particularly within the Mediterranean region, where carob trees hold considerable historical and ecological significance [13]. Numerous studies have explored the genetic and morphological diversity of carob trees in different regions, whereas there is a considerable lack of information pertaining to the germplasm found in Algeria [2,4,6,16,18,19,42].

The morphological characterization presented in this study revealed a discrete differentiation among the Algerian carob accessions. The variability among the accessions was driven by seed yield, pulp percentage, number of seeds, and seed weight. This aspect is particularly important since most of the economic interest for the species is nowadays given by the seeds whose flour (Carob Bean Gum, CBG) is an appreciated thickener used in the food industry. For the above reason, the search for accessions displaying higher seed yield and seed weight is valuable. The clustering patterns observed in the morphological characterization, particularly the distinct grouping of accessions from the Blida region, suggest an association between regional origin and carob morphology. These findings are consistent with those of Smaili et al., 2024 [6], who noted that environmental factors significantly affect the morphological traits of carob trees and emphasized the role of local adaptation in shaping the morphological diversity of carob. The distinct clustering observed among accessions from different regions further supports the hypothesis that local environmental factors or geographical isolation play a key role in driving these morphological adaptations; the moderate morphological differentiation that emerges from the obtained results highlights this species’ ability to adapt to various ecological contexts. The results confirm this tree crop′s strong adaptability to various bioclimatic conditions. As demonstrated in this study, such patterns of differentiation are fundamental for designing conservation strategies and improving the selection of desirable traits in breeding programs, which aim to enhance productivity and resilience, as demonstrated in other studies [5,6,22]. In conclusion, the morphological analyses revealed a moderate level of variability primarily influenced by the geographical regions of origin, with even greater differentiation observed at the individual level. However, except for certain pod-related traits (pod length and pod width), bioclimatic regions had a limited impact on morphological variation. This finding underscores the remarkable adaptability of this species to diverse environmental conditions.

The genetic analysis, which was based on both nuclear and chloroplast SSR markers, provided valuable insights into the genetic diversity and structure of the Algerian carob collection. The nuclear SSR analysis identified 37 alleles, with a mean number of alleles per locus of 3.81 and a mean genotypic diversity of 5.36, reflecting a moderate level of genetic diversity. The AT15 marker exhibited the highest allelic and genotypic variability and can thus be considered a key marker for distinguishing among different genotypes. Nevertheless, it is important to note that the recorded PIC value was generally below 0.7. This result can be primarily attributed to the nature of the studied material, as all analyzed accessions belong to native Algerian carob tree accessions, which inherently exhibit low genetic variability. Additionally, the limited sample size may have further contributed to the observed low polymorphism. Despite this, the findings are consistent with the work of Di Guardo et al. (2019), who, using the same approach on a larger sample of 215 accessions, not only successfully conducted a population analysis but also thoroughly validated the markers employed in this study [19]. The relatively low genetic variability observed in the chloroplast genome, as reflected by the high Simpson index (0.85–0.86), is typical for plastid markers [43], which are maternally inherited and in haploid condition. Despite the low variability, the chloroplast SSRs provided meaningful insights into the genetic relationships among the carob accessions, as demonstrated by other studies that consider the chloroplast genome to integrate the genomic studies [19,44,45].

The phylogenetic analysis revealed that the lowest genetic distances were between closely related accessions from the same geographic region, such as those from Guelma (sample ID 124 and 224) and Jijel (sample ID 118 and 218). In contrast, accessions from Souk Ahras and Bordj Bou Arréridj exhibited more significant genetic differentiation, indicating the presence of distinct genetic lineages. Moreover, this analysis has identified a distinct cluster of accessions differentiated by bioclimatic areas, with 13 accessions from humid and sub-humid areas exhibiting clear divergence from accessions in other bioclimatic origins. Building on these findings, we further conducted structure analyses using bioclimatic areas as strata to gain deeper insights into the genetic differentiation patterns.

The minimum spanning network (MSN) analysis provided further evidence of genetic structuring in relation to bioclimatic zones, with accessions from the semi-arid, humid, arid, and sub-humid regions forming bioclimatic area genetic flow patterns. This bioclimatic area pattern of genetic differentiation reinforces the idea that carob accessions are adapted to specific environmental conditions, as discussed by Smaili et al. (2024) [6]. The network revealed that specific accessions, such as those from the humid region, acted as central hubs linking multiple accessions from different ecological zones, highlighting potential genetic exchange between regions. These findings suggest that while genetic isolation may occur at smaller scales, there is still significant gene flow across bioclimatic zones, possibly mediated by factors such as human activity, seed movement, or the dispersal capacity of carob trees [46].

The DAPC and BIC analysis identified five distinct genetic clusters, which corresponded well with the bioclimatic zones of origin. This high-resolution genetic structure analysis is consistent with previous studies, which reported that carob tree accessions exhibit strong genetic differentiation based on their adaptation to specific environmental conditions [19]. The results from the DAPC and BIC analysis suggest that the bioclimatic conditions of each region highly influence the genetic structure of the Algerian carob collection. As observed in the structure analysis, the strong genetic cohesion within each bioclimatic zone further emphasizes the importance of bioclimatic adaptation in shaping the genetic diversity of carob tree accessions. These findings suggest that there is a significant gene structure similarity across bioclimatic areas, as expected and observed in other species [47,48]; the humid and sub-humid regions, as well as the arid and semi-arid regions, showed more remarkable similarity in genetic structure respectively. A Mantel test was performed to evaluate the potential correlation between the morphological and genetic distance matrices. The results did not support the hypothesis, likely due to the methodology used for genotypic analysis, as SSR markers tend to have a low level of coverage. Furthermore, the limited size of the sample examined may have also contributed to these inconsistencies.

The amalgamation of these disparate computational approaches for the genetic analysis of the Algerian carob collection contributes to a comprehensive understanding of this species′ genetic diversity, structure, and adaptive potential. The findings underscore the importance of bioclimatic factors in shaping genetic relationships. Although the minimum spanning network analyses contributed to visualizing the distance between the different accessions studied and the genetic flow among the different bioclimatic regions, the genetic structure analysis allowed for the identification and definition of the accession by bioclimatic regions.

Similar studies have been conducted on various species, such as sweet chestnut and olive, to clarify the genetic structure and morphological diversity of germplasm collections within a defined region. For instance, Marinoni et al. (2013) conducted both genetic characterization, using 10 SSR markers, and morphological analysis on 68 sweet chestnut accessions. Their findings revealed that morphological traits effectively distinguished the four gene pools identified in the germplasm, although only a few traits proved useful in discriminating cultivars. Cultivars tended to cluster within a primary gene pool based on their growing area [49]. Similarly, Sorkheh et al. (2016) examined 200 olive trees across four regions, using morphological traits and SSR markers. Significant variation was observed in traits like fruit weight and stone width. SSR markers revealed greater genetic variability than morphological traits, emphasizing their effectiveness in detecting genetic diversity. The inconsistency between molecular and morphological data suggests that each marker system captures different aspects of genetic variability [50].

Moreover, SSR markers were implemented to analyze the genetic relationship and structure in olive tree cultivated in southern Italy, revealing the clustering of the 68 olive cultivars according to geographic grouping [51]

These findings highlight the importance of integrating genetic and morphological analyses, as also demonstrated in the present study on Algerian carob tree, where a combination of both approaches provided a comprehensive understanding of the species′ genetic diversity and adaptation. In a context where the genetic characterization of this species in Algeria has been remarkably limited, this study not only corroborates previous research on carob tree genetics but also lays a solid foundation for future investigations. By providing valuable insights into the genetic diversity and structure of Algerian carob accessions, it contributes essential knowledge for developing sustainable conservation strategies and breeding programs. Genetic characterization of plant material, as conducted in this study, is crucial for conservation plans. Accurate plant material identification enhances the management of genetic resources. In the case of in situ conservation, understanding the genetic profiles of accessions helps monitor the genetic diversity of natural populations and develop strategies to protect neglected varieties that contribute to the genetic heritage. For ex situ conservation strategies, employing a genetic approach to characterize the plant material is more effective and reduces the likelihood of errors when establishing germplasm collections. This, in turn, supports breeding and nursery activities [52]. These findings are particularly relevant for enhancing carob cultivation in Algeria, guiding both genetic resource management and the selection of superior accessions for agricultural and industrial purposes.

## 5. Conclusions

In conclusion, this study provides significant insights into the genetic diversity, population structure, and breeding potential of the genetic resources of the carob tree in the specific context of a large Mediterranean area region in Algeria. The results highlight the importance of Algerian carob accessions in displaying genetic diversity and offer valuable information for the sustainable exploitation of carob genetic resources. The identification of genetically distinct populations also opens up opportunities for the introgression of beneficial traits. Future research should focus on the propagation of selected promising accessions, as well as their conservation and evaluation in different areas, so that valuable traits could be exploited both for cultivation and breeding.

## Figures and Tables

**Table 1:** List of carob accessions included in this study and geographical and ecological characteristics of their sampling area.

Sample ID	Regions	Latitude	Longitude	Altitude (m)	Bioclimate	Type
116	Algiers	36°43′17” N	3°09′01” E	45	Humid	Cultivated
123	Annaba	36°55′18′′ N	7°44′00′′ E	241	Humid	Wild
223	Annaba	36°55′20′′ N	7°43′54′′ E	287	Humid	Wild
323	Annaba	36°55′23′′ N	7°43′53′′ E	294	Humid	Wild
106	Bejaia	36°34′59′′ N	5°25′59′′ E	289	Humid	Wild
206	Bejaia	36°35′07′′ N	5°25′58′′ E	340	Humid	Wild
107	Biskra	34°50′37′′ N	5°44′04′′ E	107	Arid	Cultivated
207	Biskra	34°50′36′′ N	5°44′05′′ E	108	Arid	Cultivated
109	Blida	36°31′54′′ N	2°55′43′′ E	112	Sub-Humid	Cultivated
209	Blida	36°31′54′′ N	2°55′44′′ E	113	Sub-Humid	Cultivated
109n	Blida	36°31′54′′ N	2°55′45′′ E	113	Sub-Humid	Cultivated
209n	Blida	36°31′52′′ N	2°55′42′′ E	113	Sub-Humid	Cultivated
134	Bordj Bou Arréridj	35°55′35′′ N	4°44′59′′ E	995	Semi-Arid	Cultivated
234	Bordj Bou Arréridj	35°55′27′′ N	4°45′5′′ E	982	Semi-Arid	Wild
110	Bouïra	36°35′28′′ N	3°34′30′′ E	105	Semi-Arid	Wild
210	Bouïra	36°35′43′′ N	3°34′34′′ E	108	Semi-Arid	Wild
135	Boumerdès	36°49′36′′ N	3°50′21′′ E	268	Sub-Humid	Cultivated
136	El Taref	36°39′21′′ N	7°47′36′′ E	35	Sub-Humid	Cultivated
336	El Taref	36°39′22′′ N	7°47′45′′ E	33	Sub-Humid	Cultivated
236	El Taref *	36°39′22′′ N	7°47′45′′ E	33	Sub-Humid	Cultivated
124	Guelma	36°18′28′′ N	7°35′24′′ E	812	Sub-Humid	Wild
224	Guelma	36°18′23′′ N	7°35′14′′ E	846	Sub-Humid	Wild
118	Jijel	36°39′49′′ N	6°16′35′′ E	62	Humid	Wild
218	Jijel	36°39′48′′ N	6°16′41′′ E	86	Humid	Wild
104	Oum El Bouaghi	35°52′02′′ N	7°08′18′′ E	901	Semi-Arid	Cultivated
204	Oum El Bouaghi	35°52′04′′ N	7°08′17′′ E	903	Semi-Arid	Cultivated
119	Sétif	36°32′31′′ N	5°04′49′′ E	931	Semi-Arid	Wild
121	Skikda	36°43′22′′ N	7°16′29′′ E	45	Humid	Cultivated
221	Skikda	36°43′22′′ N	7°16′29′′ E	45	Humid	Cultivated
321	Skikda	36°43′22′′ N	7°16′29′′ E	45	Humid	Cultivated
141	Souk Ahras	36°23′31″ N	8°21′41″ E	490	Semi-Arid	Wild
241	Souk Ahras	36°24′28” N	8°21′55” E	261	Semi-Arid	Wild
142	Tipaza	36°31′12′′ N	2°29′13′′ E	73	Sub-Humid	Cultivated
242	Tipaza	36°31′12′′ N	2°29′13′′ E	73	Sub-Humid	Cultivated
342	Tipaza	36°31′12′′ N	2°29′12′′ E	73	Sub-Humid	Cultivated
115	Tizi Ouzou	36°45′35′′ N	4°22′12′′ E	339	Humid	Wild
215	Tizi Ouzou	36°45′33′′ N	4°22′15′′ E	347	Humid	Wild
315	Tizi Ouzou	36°45′33′′ N	4°22′15′′ E	347	Humid	Wild
415	Tizi Ouzou	36°53′54′′ N	4°17′51′′ E	86	Humid	Wild

* Hermaphroditic cultivar.

**Table 2:** Polymorphism information of nuclear markers considering the whole set of alleles.

Nuclear SSR	Alleles	Genotypes	No. of Obs.	*H_e_*	*H_o_*	*PIC*	Simpson Index (D)
CTTT7	2	2	38	0.19	0.21	0.17	0.2
AT15	8	11	35	0.63	0.51	0.59	0.62
2GA12	4	7	38	0.45	0.39	0.40	0.46
AT9	5	9	38	0.61	0.24	0.55	0.62
GCT6	3	5	37	0.36	0.38	0.31	0.37
TA5TG6	3	4	38	0.37	0.37	0.33	0.36
TA7	2	4	38	0.43	0.45	0.33	0.42
TTA7	7	9	38	0.60	0.82	0.52	0.59

*H_e_*: expected heterozygosity; *H_o_*: observed heterozygosity, *PIC*: polymorphic information content.

**Table 3:** Polymorphic information content of chloroplastic markers.

Chloroplastic SSR	Alleles	No. of Obs.	Simpson Index (D)
ccSA-ndhd	3	36	0.85
rpl32-TrnL	2	38	0.85
PsbD-Trn	3	38	0.86

## Data Availability

Data are contained within the article and Supplementary Materials.

## Supplementary Materials

The following supporting information can be downloaded at: www.mdpi.com/article/10.3390/plants14070990/s1, Table S1: Results of the morphological analyses carried out on the pods and seeds encompassing the Algerian carob accessions of the present study; Table S2: Results of the morphological analyses carried out on the pods and seeds encompassing the Algerian carob accessions across the four bioclimatic area; Table S3: SSR profiles of the Algerian carob accession of the present research; Figure S1: Genotypic accumulation curve computed in the SSR dataset of the Algerian carob collection. On the x-axis is the minimum number of loci to discriminate a sample, and on the y-axis are the multilocus genotypes observed; Figure S2: Distribution of genetic variations at different hierarchical levels (AMOVA). The top panel represents variations within samples, showing the spread of genetic diversity among individuals. The middle panel illustrates variations between samples, highlighting individual differences across samples. The bottom panel depicts variations between bioclimatic populations (Pop), reflecting genetic differentiation among populations, with an outlier on the right. Histograms show the frequency distribution of variation, and outliers are marked with black rhombi.
