# Supplementary material for: Comprehensive Genetic and Morphological Analysis of Algerian Carob (Ceratonia siliqua L.) Accessions
Source: Plants (Basel). 2025 Mar 21;14(7):990. doi: 10.3390/plants14070990 (PMC11990269; doi:10.3390/plants14070990)
Supplement: Supplementary file 1 [file plants-14-00990-s001.zip › plants-3515292-supplementary.pdf]

Supplementary Table S1: Results of the morphological analyses carried out on the pods and seeds encompassing the Algerian carob accessions of the present study.

Accession	Pods								Seeds			
	Length (mm)	Width (mm)	Thickness (mm)	Weight (g)	Pulp weight (g)	Number of seeds	% of Seed	% of pulp	Length (mm)	Width (mm)	Thickness (mm)	Weight per pod (g)
0116	145 ± 6.4	25.27 ± 0.8	9.35 ± 0.43	20.68 ± 1.84	17.61 ± 1.68	13.27 ± 1.31	14.86 ± 1.37	85.14 ± 1.37	10.75 ± 0.34	7.45 ± 0.32	3.91 ± 0.25	3.07 ± 0.32
0123	141 ± 11.3	18.07 ± 1.68	8.46 ± 1.20	10.71 ± 2.26	9.18 ± 2.05	8.43 ± 1.94	14.36 ± 3.41	85.64 ± 3.41	9.71 ± 0.97	6.69 ± 0.40	4.37 ± 0.24	1.52 ± 0.44
0223	152 ± 10.5	23.23 ± 1.65	7.08 ± 0.89	9.99 ± 2.83	7.98 ± 2.84	10.73 ± 1.60	21.43 ± 6.07	78.57 ± 6.07	9.91 ± 0.40	7.35 ± 0.32	3.64 ± 0.19	2.01 ± 0.42
0323	146 ± 12.9	20.05 ± 1.13	7.59 ± 0.70	11.99 ± 1.33	9.55 ± 1.00	12.07 ± 1.87	20.32 ± 1.77	79.68 ± 1.77	9.33 ± 0.37	7.49 ± 0.37	4.17 ± 0.24	2.45 ± 0.40
0106	157 ± 9.5	19.98 ± 1.05	5.28 ± 0.56	6.29 ± 0.67	4.14 ± 0.44	12.13 ± 1.48	34.08 ± 3.78	65.92 ± 3.78	9.18 ± 0.30	7.28 ± 0.30	3.65 ± 0.20	2.15 ± 0.38
0206	124 ± 10.3	19.98 ± 1.29	6.26 ± 0.77	5.03 ± 0.67	3.75 ± 0.54	8.93 ± 1.51	25.49 ± 2.96	74.51 ± 2.96	8.70 ± 0.32	6.97 ± 0.30	3.25 ± 0.29	1.28 ± 0.22
0107	146 ± 11.1	14.14 ± 1.48	5.86 ± 0.66	7.08 ± 1.19	5.16 ± 1.08	12.47 ± 1.83	27.57 ± 4.13	72.43 ± 4.13	8.59 ± 0.48	6.69 ± 0.36	3.71 ± 0.43	1.92 ± 0.25
0207	155 ± 21.0	17.41 ± 2.23	6.78 ± 1.08	10.07 ± 2.97	8.12 ± 2.86	12.50 ± 1.83	20.63 ± 5.56	79.37 ± 5.56	8.50 ± 0.49	6.62 ± 0.40	4.11 ± 0.38	1.94 ± 0.29
0109	174 ± 22.8	26.52 ± 1.17	10.90 ± 1.02	29.79 ± 5.01	27.29 ± 4.72	11.97 ± 2.28	8.48 ± 1.33	91.52 ± 1.33	8.90 ± 0.32	7.37 ± 0.22	4.56 ± 0.21	2.51 ± 0.48
0209	201 ± 21.0	26 ± 1.8	11.22 ± 0.85	37.23 ± 7.92	34.19 ± 7.59	14.03 ± 1.79	8.35 ± 1.12	91.65 ± 1.12	8.93 ± 0.30	7.34 ± 0.18	4.62 ± 0.22	3.04 ± 0.40
0109n	162 ± 26.0	26.07 ± 1.7	12.06 ± 0.71	27.53 ± 7.44	25.59 ± 7.01	9.43 ± 2.45	7.10 ± 1.44	92.90 ± 1.44	8.81 ± 0.35	7.32 ± 0.37	4.59 ± 0.22	1.94 ± 0.56
0209n	173 ± 29.6	24.91 ± 1.6	11.54 ± 0.68	29.50 ± 7.99	27.20 ± 7.53	11.03 ± 2.58	7.95 ± 1.20	92.05 ± 1.20	8.94 ± 0.43	7.35 ± 0.29	4.63 ± 0.22	2.30 ± 0.55
0134	130 ± 6.5	17.6 ± 0.7	5.65 ± 0.48	6.35 ± 0.63	4.89 ± 0.54	10.93 ± 0.58	23.06 ± 1.51	76.94 ± 1.51	7.45 ± 0.15	6.36 ± 0.14	4.08 ± 0.26	1.46 ± 0.13
0234	143 ± 13.7	17.1 ± 0.7	4.16 ± 0.33	5.32 ± 0.76	3.80 ± 0.55	11.37 ± 1.97	28.62 ± 2.53	71.38 ± 2.53	8.86 ± 0.33	6.42 ± 0.26	3.45 ± 0.10	1.53 ± 0.27
0110	114 ± 9.3	17.37 ± 1	4.46 ± 0.45	5.09 ± 0.62	3.40 ± 0.53	11.83 ± 1.39	33.43 ± 3.92	66.57 ± 3.92	8.91 ± 0.38	6.59 ± 0.33	3.68 ± 0.22	1.69 ± 0.20
0210	122 ± 7.0	17.2 ± 1	5.14 ± 0.34	5.28 ± 0.43	3.63 ± 0.34	12.13 ± 0.97	31.28 ± 2.16	68.72 ± 2.16	9.15 ± 0.21	5.84 ± 0.17	3.70 ± 0.14	1.65 ± 0.15
0135	141 ± 17.3	23.5 ± 2	6.55 ± 0.86	10.64 ± 2.94	9.02 ± 2.53	8.90 ± 2.29	15.29 ± 2.18	84.71 ± 2.18	9.05 ± 0.53	6.78 ± 0.43	4.13 ± 0.60	1.62 ± 0.47
0136	132 ± 16.7	19.28 ± 1	6.29 ± 0.56	6.41 ± 1.47	5.15 ± 1.15	7.17 ± 1.91	19.55 ± 2.25	80.45 ± 2.25	9.13 ± 0.33	7.40 ± 0.33	3.78 ± 0.23	1.26 ± 0.35
0236	147 ± 9.8	25 ± 1.	5.99 ± 0.74	8.35 ± 0.89	6.78 ± 0.74	9.13 ± 1.38	18.77 ± 1.57	81.23 ± 1.57	9.61 ± 0.31	7.52 ± 0.27	3.25 ± 0.16	1.57 ± 0.20
0336	146 ± 9.8	21.6 ± 2	6.97 ± 0.4	10.08 ± 1.31	8.31 ± 1.20	9.60 ± 1.61	17.66 ± 2.99	82.34 ± 2.99	10.44 ± 0.38	7.74 ± 0.26	3.39 ± 0.17	1.77 ± 0.32
0124	146 ± 12.6	18.74 ± 1	5.22 ± 0.42	8.60 ± 1.14	7.57 ± 1.11	10.50 ± 1.48	12.03 ± 3.57	87.97 ± 3.57	9.27 ± 0.36	6.12 ± 0.53	2.55 ± 0.46	1.03 ± 0.30
0224	160 ± 18.9	19.6 ± 1.1	6.99 ± 0.59	12.21 ± 2.24	10.97 ± 2.03	8.73 ± 1.64	10.17 ± 1.71	89.83 ± 1.71	8.52 ± 0.40	6.72 ± 0.35	3.52 ± 0.25	1.24 ± 0.29
0118	138 ± 9.6	17.82 ± 1	4.86 ± 0.43	6.77 ± 0.90	5.39 ± 0.82	9.97 ± 1.83	20.52 ± 4.03	79.48 ± 4.03	8.42 ± 0.31	6.28 ± 0.26	3.62 ± 0.26	1.38 ± 0.28
0218	128 ± 17.4	20 ± 1.79	5.87 ± 0.56	7.99 ± 1.94	6.41 ± 1.67	9.93 ± 2.13	20.08 ± 2.66	79.92 ± 2.66	9.32 ± 0.55	7.02 ± 0.33	3.47 ± 0.33	1.58 ± 0.34
0104	166 ± 13.8	20.8 ± 1	5.96 ± 0.45	12.97 ± 1.71	11.27 ± 1.49	11.77 ± 2.03	13.14 ± 1.81	86.86 ± 1.81	8.83 ± 0.31	6.71 ± 0.27	3.69 ± 0.20	1.71 ± 0.34
0204	171 ± 16.0	22.4 ± 1.3	8.85 ± 1.24	20.58 ± 3.77	19.11 ± 3.63	9.27 ± 1.51	7.24 ± 1.27	92.76 ± 1.27	9.50 ± 0.31	7.19 ± 0.15	3.27 ± 0.20	1.47 ± 0.26
0119	171 ± 13.0	24.4 ± 0.8	6.08 ± 0.55	15.88 ± 1.55	13.47 ± 1.29	11.47 ± 1.91	15.14 ± 1.89	84.86 ± 1.89	9.75 ± 0.33	7.47 ± 0.29	3.98 ± 0.24	2.41 ± 0.41
0121	122 ± 11.0	19 ± 0.95	6.37 ± 0.47	9.25 ± 1.39	7.76 ± 1.25	10.47 ± 1.50	16.30 ± 2.71	83.70 ± 2.71	9.45 ± 0.44	6.93 ± 0.42	3.26 ± 0.28	1.50 ± 0.27
0221	152 ± 21.3	23.3 ± 1.3	7.99 ± 0.95	14.37 ± 3.55	12.17 ± 3.17	10.30 ± 2.17	15.59 ± 2.70	84.41 ± 2.70	10.77 ± 0.30	7.68 ± 0.21	3.62 ± 0.16	2.20 ± 0.50
0321	135 ± 14.1	25.1 ± 1.8	8.96 ± 1.61	18.27 ± 4.53	16.75 ± 4.33	8.23 ± 1.83	8.59 ± 2.14	91.41 ± 2.14	9.64 ± 0.36	6.86 ± 0.20	3.97 ± 0.20	1.52 ± 0.35
0141	113 ± 13.3	21.7 ± 1.2	7.36 ± 0.57	9.98 ± 1.91	8.40 ± 1.51	8.87 ± 2.06	15.67 ± 2.73	84.33 ± 2.73	9.72 ± 0.21	6.97 ± 0.38	3.97 ± 0.41	1.59 ± 0.47
0241	128 ± 19.0	15.9 ± 2.3	7.46 ± 1.43	11.37 ± 2.48	9.92 ± 2.24	7.70 ± 2.55	12.69 ± 4.60	87.31 ± 4.60	9.96 ± 0.24	7.13 ± 0.31	3.54 ± 0.31	1.45 ± 0.58
0142	191 ± 12.6	23.4 ± 0.8	5.89 ± 0.50	11.70 ± 1.82	9.63 ± 1.79	11.87 ± 1.33	18.07 ± 3.31	81.93 ± 3.31	10.54 ± 0.46	6.93 ± 0.34	3.22 ± 0.23	2.07 ± 0.27
0242	182 ± 15.2	22.4 ± 0.8	5.82 ± 0.69	10.84 ± 1.10	8.80 ± 0.85	12.13 ± 1.74	18.72 ± 1.66	81.28 ± 1.66	10.16 ± 0.42	6.86 ± 0.22	3.29 ± 0.12	2.04 ± 0.32
0342	169 ± 14.0	22 ± 0.86	5.52 ± 0.70	10.05 ± 1.58	8.45 ± 1.26	10.20 ± 1.79	15.79 ± 2.10	84.21 ± 2.10	10.10 ± 0.31	6.63 ± 0.41	3.21 ± 0.26	1.60 ± 0.40
0115	146 ± 11.0	22.1 ± 2.1	7.37 ± 0.77	13.68 ± 2.52	11.44 ± 2.47	11.20 ± 1.92	16.80 ± 4.17	83.20 ± 4.17	9.74 ± 0.67	7.27 ± 0.30	3.97 ± 0.20	2.24 ± 0.47
0215	144 ± 10.2	21.7 ± 1	7.54 ± 0.44	14.71 ± 1.69	12.68 ± 1.52	11.00 ± 1.51	13.82 ± 1.36	86.18 ± 1.36	9.20 ± 0.30	7.00 ± 0.20	4.01 ± 0.21	2.03 ± 0.26
0315	112 ± 13.3	22.2 ± 1.5	7.57 ± 0.62	10.66 ± 2.06	9.10 ± 1.71	8.07 ± 1.87	14.50 ± 2.15	85.50 ± 2.15	9.04 ± 0.47	6.97 ± 0.25	4.19 ± 0.21	1.56 ± 0.43
0415	171 ± 10.5	21.4 ± 0.7	8.77 ± 0.59	17.73 ± 1.81	14.51 ± 1.56	13.67 ± 1.65	18.19 ± 1.39	81.81 ± 1.39	10.64 ± 0.30	7.65 ± 0.22	4.57 ± 0.18	3.22 ± 0.36

The data represents the mean value ± the standard deviation.

Supplementary Table S2: Results of the morphological analyses carried out on the pods and seeds encompassing the Algerian carob accessions across the four bioclimatic area.

Bioclimatic region	Pod leng	Pod width	Pod thickness	Pod weight	Seeds count	Seed Weight	Pulp weigh	Seed length	Seed width	Seed thickness	Seed Yield	Pulp Percentage
**Arid**	151±5.0^{ab}	15.8±1.6^{c}	6.3±0.5	8.6±1.5	12.5±0.02	1.9±0.01	6.6±1.5	8.5±0.05	6.7±0.03	3.9±0.2	24.1±3.5	75.9±3.5
**Humid**	141±4.0^{b}	21.3±0.6^{ab}	7.3±0.3	11.9±1.2	10.6±0.4	2±0.1	9.9±1.1	9.6±0.2	7.1±0.1	3.8±0.1	18.3±1.5	81.7±1.5
**Semi Arid**	140±8.0^{b}	19.4±1^{bc}	6.1±0.5	10.3±1.8	10.6±0.5	1.7±0.1	8.6±1.8	9.1±0.2	6.7±0.2	3.7±0.1	20±3.1	79.9±3.2
**Sub Humid**	164±5.0^{a}	23.1±0.7^{a}	7.8±0.7	16.4±2.9	10.4±0.5	1.8±0.1	14.5±2.8	9.4±0.2	7.1±0.1	3.7±0.2	13.7±1.3	86.3±1.3
**Sign.**	*	**	N.S.	N.S.	N.S.	N.S.	N.S.	N.S.	N.S.	N.S.	N.S.	N.S.

The data represents the mean value ± the standard deviation. The significance level (Sign.), determined by the ANOVA p-value and assessed using the Tukey test as a post hoc analysis, is denoted as * for $p < 0.05$ and ** for $p < 0.01$, while N.S. indicates non-significant results.

Supplementary Table S3: SSR profiles of the Algerian carob accession of the present research.

Accessions	Nuclear SSR markers								Chloroplastic SSR markers		
	**CTTT7**	**AT15**	**GA12**	**AT9**	**GCT6**	**TA5TG6**	**TA7**	**TTA7**	**ccSA-ndhd**	**_rpl32-TrnL**	**PsbD_Trn**
104	210/210	162/173	110/114	174/174	183/183	272/276	298/300	233/242	446	257	407
106	210/210	172/176	114/114	176/176	183/189	272/272	298/298	233/242	447	256	387
107	210/210	168/172	114/114	174/176	183/189	272/276	298/298	233/242	446	256	407
109	202/210	162/181	110/124	172/172	180/183	272/272	298/298	233/242	446	257	408
110	210/210	**NA**	114/114	174/174	183/189	272/272	298/298	233/242	446	257	408
115	210/210	162/176	110/114	172/176	183/189	272/276	298/298	230/233	446	257	408
116	210/210	162/162	114/124	161/174	183/183	272/272	298/300	233/233	446	257	408
118	210/210	172/172	112/112	176/194	183/183	272/276	298/300	233/242	447	256	387
119	202/210	162/162	110/114	176/176	183/183	272/272	298/300	233/242	446	257	407
121	210/210	162/168	114/124	174/174	183/183	272/272	298/298	233/233	456	257	407
123	210/210	162/162	114/114	172/172	183/183	272/272	298/298	233/263	446	257	407
124	210/210	162/168	114/114	174/176	180/183	270/276	298/298	233/242	447	256	387

134	202/210	162/176	110/114	174/174	183/183	272/276	300/300	233/233	446	257	407
135	210/210	162/162	110/112	174/174	183/183	272/272	298/300	233/242	446	257	407
136	210/210	174/174	114/114	174/174	183/189	272/272	300/300	233/242	447	256	387
141	210/210	172/176	114/114	172/176	183/183	272/272	300/300	233/242	446	257	407
142	210/210	162/162	112/114	176/176	183/183	272/272	298/298	233/242	447	256	387
204	202/210	162/162	110/110	172/172	183/183	272/272	298/300	233/242	446	257	407
206	210/210	162/162	110/114	174/174	183/183	272/276	298/300	233/242	446	257	407
207	210/210	162/162	114/114	174/174	183/183	270/276	298/298	233/242	446	257	407
209	210/210	NA	114/114	174/174	183/189	272/272	298/300	242/242	447	256	407
210	210/210	162/168	110/114	174/174	183/183	272/272	298/298	233/233	446	257	407
215	210/210	162/162	114/114	174/174	183/183	270/276	298/300	233/242	456	257	408
218	210/210	162/172	112/114	174/174	183/183	272/276	298/298	233/263	447	256	387
221	210/210	162/162	114/114	174/174	189/189	272/272	298/300	233/242	447	256	387
223	210/210	NA	114/114	174/174	183/183	272/272	298/298	233/263	447	256	387
224	210/210	162/170	114/114	194/194	180/183	270/272	298/298	233/233	447	256	387
234	210/210	162/176	114/114	172/172	183/183	272/272	298/298	224/242	NA	257	407
236	202/210	162/162	110/114	174/174	183/183	272/272	298/298	233/233	446	257	407
241	202/210	162/172	114/114	172/176	183/183	272/276	298/300	227/233	446	257	407
242	210/210	174/176	114/114	174/174	183/189	272/272	298/300	230/242	447	256	387
315	202/210	162/162	114/114	172/176	183/183	272/276	298/298	230/242	446	257	407
321	210/210	174/176	114/114	174/174	183/189	272/272	298/300	233/242	447	256	387
323	202/210	162/162	110/114	172/176	0/0	270/272	298/300	233/236	446	257	407

336	210/210	162/162	114/114	176/176	183/189	272/272	298/298	233/242	0	257	407
342	210/210	174/176	114/114	174/174	183/189	272/272	298/300	233/242	447	256	387
415	210/210	162/162	110/114	174/174	183/183	272/272	298/300	233/242	446	257	407
0109n	210/210	174/176	114/114	174/174	183/189	272/272	298/300	233/242	447	256	387
0209n	202/210	162/172	110/124	172/172	180/183	272/272	298/298	233/242	446	257	407

NA: non-genotyped marker in the dataset.

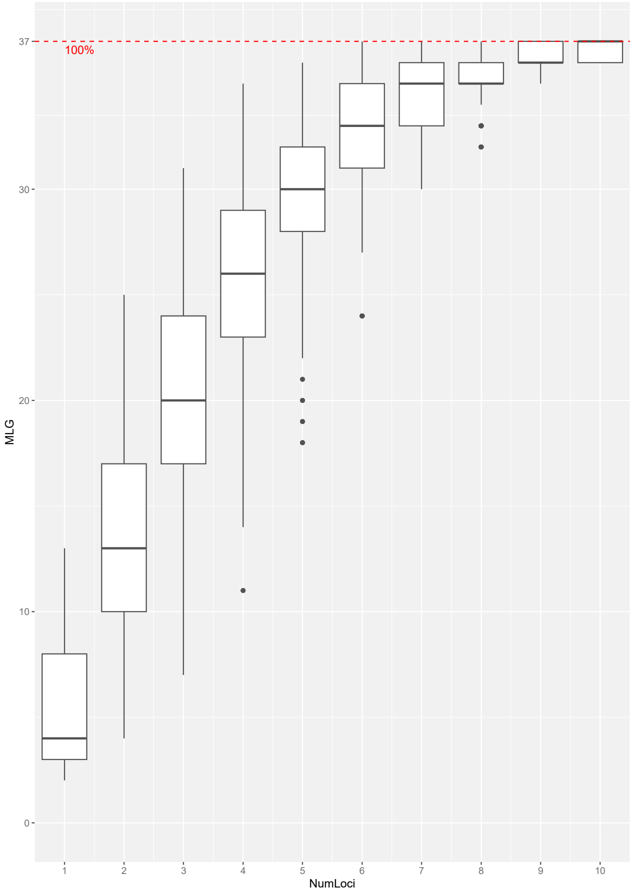

Supplementary Figure S1: Genotypic accumulation curve computed in the SSR dataset of the Algerian carob collection. On the x-axis is the minimum number of loci to discriminate a sample, and on the y-axis are the multilocus genotypes observed.

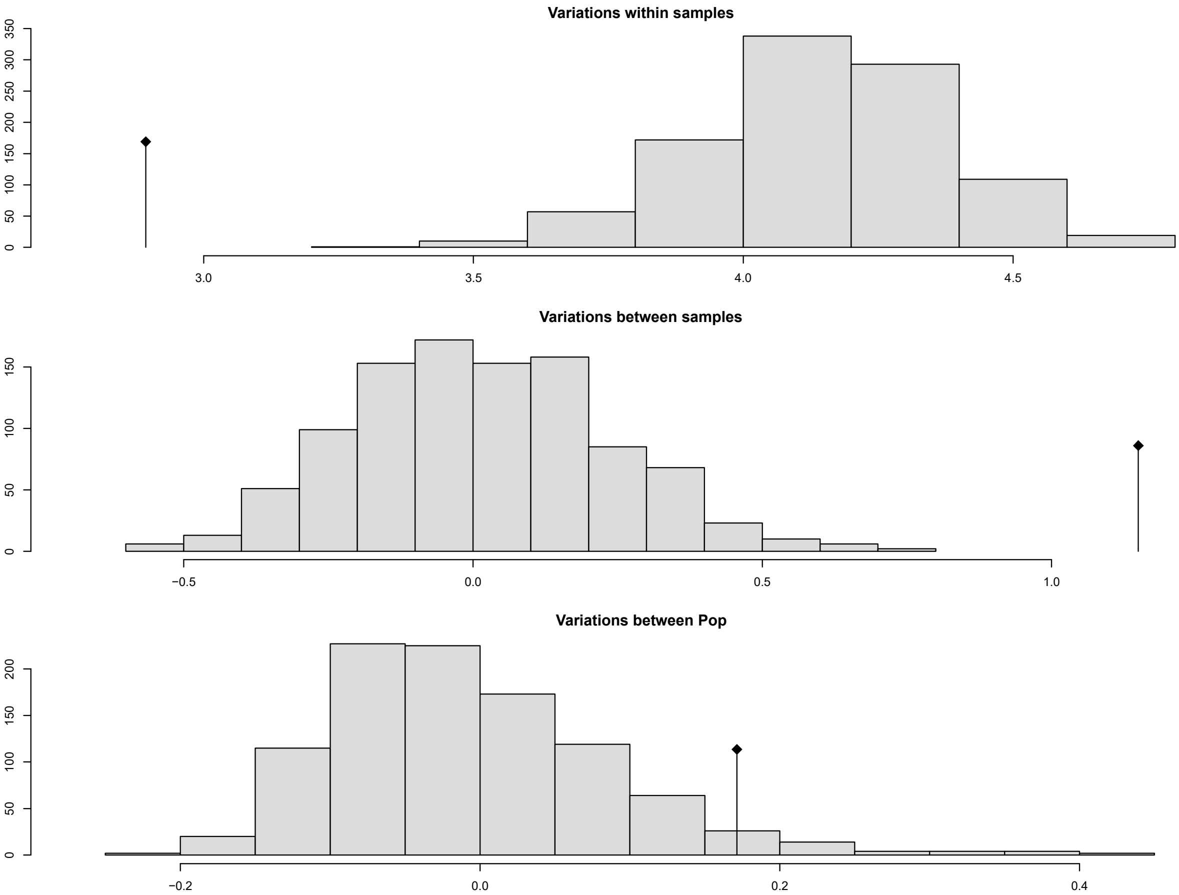

Supplementary Figure S2: Distribution of genetic variations at different hierarchical levels (AMOVA). The top panel represents variations within samples, showing the spread of genetic diversity among individuals. The middle panel illustrates variations between samples, highlighting individual differences across samples. The bottom panel depicts variations between bioclimatic populations (Pop), reflecting genetic differentiation among populations, with an outlier on the right. Histograms show the frequency distribution of variation, and outliers are marked with black rhombi.
